# Utility of combined dynamic chest radiology in chronic thromboembolic pulmonary disease

**DOI:** 10.1093/ehjcr/ytae019

**Published:** 2024-01-09

**Authors:** Koshi Setoyama, Yoshiko Hayashida, Takatoshi Aoki

**Affiliations:** The Second Department of Internal Medicine, University of Occupational and Environmental Health, 1-1 Iseigaoka, Yahatanishi-ku, 807-8555 Kitakyusyu, Japan; Department of Radiology, University of Occupational and Environmental Health, Kitakyusyu, Japan; Department of Radiology, University of Occupational and Environmental Health, Kitakyusyu, Japan

A 74-year-old woman was referred to our hospital with a chief complaint of dyspnoea on exertion and was diagnosed with chronic thromboembolic pulmonary disease (CTEPD). The patient displayed a mean pulmonary artery pressure of 32 mmHg. *Panels A* and *B* show pulmonary perfusion/ventilation scintigraphy, which suggests blood flow deficit in the right middle to lower lobes and left apex. *Panel C* shows pulmonary angiography, which suggests avascular areas in the same segments. *Panel D* shows dynamic chest radiology (DCR) to evaluate pulmonary perfusion, thereby suggesting perfusion defect in the same segments. *Panel E* depicts high-resolution computed tomography, showing a wedge shadow (arrowhead) that is located just below the pleura in the right middle lobe and thereby demonstrates pulmonary infarction. *Panel F* shows a DCR image visualizing pulmonary expansion and motion. The area indicated by the arrow suggests poor pulmonary motion and thus insufficient expansion. These findings may reflect decreased lung mobility due to pulmonary tissue ischaemia caused by thromboembolism.

Revascularization of the area of pulmonary infarction increases the risk of complications, such as reperfusion pulmonary oedema and pulmonary artery injury. Although DCR is known to be a simple perfusion assessment tool for CTEPD patients, it can be an excellent tool to assess pulmonary viability by combining DCR with assessments of pulmonary perfusion and mobility. This method ultimately leads to an optimal treatment strategy, in particular, to assess target lesions for balloon pulmonary angioplasty in CTEPD.

**Figure:**
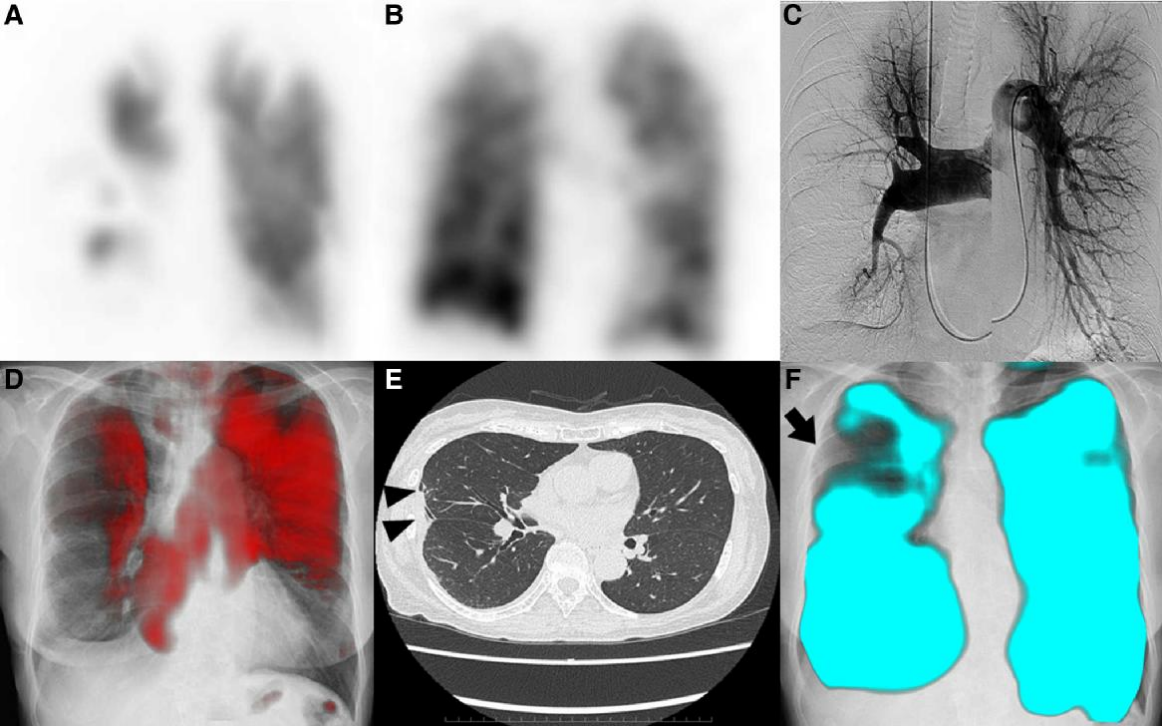


(*A*, *B*) Pulmonary perfusion/ventilation scintigraphy. (*C*) Pulmonary angiography. (*D*) Dynamic chest radiology to evaluate pulmonary perfusion. (*E*) High-resolution computed tomography. (*F*) Dynamic chest radiology image visualizing pulmonary expansion.

## Data Availability

Open access.

